# First- and second-line bevacizumab in ovarian cancer: A Belgian cost-utility analysis

**DOI:** 10.1371/journal.pone.0195134

**Published:** 2018-04-09

**Authors:** Mattias Neyt, Joan Vlayen, Stephan Devriese, Cécile Camberlin

**Affiliations:** Belgian Health Care Knowledge Centre (KCE), Brussels, Belgium; CANADA

## Abstract

**Background:**

Currently, in Belgium, bevacizumab is reimbursed for ovarian cancer patients, based on a contract between the Minister and the manufacturer including confidential agreements. This reimbursement will be re-evaluated in 2018.

**Objective:**

To support the reimbursement reassessment by calculating the cost-effectiveness of bevacizumab: (1) in addition to first-line chemotherapy; (2) in the treatment of recurrent ovarian cancer (platinum-sensitive or platinum-resistant).

**Methods:**

A health economic model has been developed for the Belgian situation according to the Belgian guidelines for economic evaluations. The lifetime Markov model was set up from the perspective of the health care payer (government and patient), including direct healthcare related costs. Results are expressed as the extra costs per quality-adjusted life year (QALY). Calculations were based on results of four international trials. Both probabilistic and one-way sensitivity analyses were performed.

**Results:**

Incremental cost-effectiveness ratios (ICERs) of first-line bevacizumab are on average 158 000/QALY (GOG-0218 trial) and 443 000/QALY (ICON7 trial). The most favourable scenario is based on the stage IV subgroup of the GOG-0218 trial (€52 000/QALY). Since subgroup findings are often exploratory and require confirmatory studies, results of the economic evaluation based on this subgroup analysis should be considered with caution. For second-line bevacizumab, ICERs are on average €587 000/QALY (OCEANS trial) and €172 000/QALY (AURELIA trial). Sensitivity analysis shows that results are most sensitive to the price of bevacizumab.

**Conclusion:**

From a health economic perspective, ICERs of bevacizumab are relatively high. The most favourable results are found for first-line treatment of stage IV ovarian cancer patients. Price reductions have a major impact on the estimated ICERs. It is recommended to take these findings into account when re-evaluating the reimbursement of bevacizumab in ovarian cancer.

## Introduction

In Belgium, ovarian cancer is the eighth most frequent female cancer and the fifth in terms of female cancer mortality.[1] In 2014, 848 women were diagnosed with ovarian cancer. The mean age at diagnosis was 66.7 years. Around 72% of all ovarian cancers are diagnosed in an advanced stage when the tumour has already spread outside the pelvic area, to the retroperitoneal lymph nodes or beyond the peritoneum (stage III-IV).[2] The global relative five-year survival is 42.6%, but only 23.8% for stage IV.[3]

In advanced stage, cytoreductive surgery and chemotherapy are recommended according to the Belgian guidelines.[4] Standard first-line chemotherapy combination is carboplatin-paclitaxel.[4] For second-line chemotherapy, this combination may be re-used in platinum-sensitive patients, and carboplatin may also be switched to cisplatin. In case of allergy to platinum-based compounds, or non-response of the tumour to platinum-based chemotherapy (platinum-refractory tumour) or in case of relapse within 6 months after this kind of chemotherapy (platinum-resistant tumour), paclitaxel alone, pegylated liposomal doxorubicin hydrochloride (PLD), topotecan or gemcitabine represent possible options.[5–7]

For epithelial ovarian, fallopian or primitive peritoneal cancer, front-line treatment with bevacizumab in stage IIIB-IIIC-IV was authorized by the European Medicines Agency on 21 September 2011. This indication was extended to recurrent platinum-sensitive cancer on 20 September 2012 and to recurrent platinum-resistant cancer on 31 July 2014.[4] In Belgium, reimbursement for epithelial ovarian, fallopian or primitive peritoneal cancer (onwards we refer to this by using the term ‘ovarian cancer’) can be allowed in three types of indications: (1) first-line treatment of stage FIGO IV ovarian cancer (in combination with carboplatin and paclitaxel for a maximum of 6 treatment cycles and then in monotherapy until either a maximum of 15 months in total, disease progression or unacceptable toxicity); (2) first recurrence of ovarian cancer in patients sensitive to platin salts who have not yet been treated with bevacizumab or other VEGF inhibitors or other agents aiming at the VEGF receptor (in combination with carboplatin and gemcitabine during 6 to maximum 10 cycles and then in monotherapy until disease progression); or (3) second-line treatment of ovarian cancer in patients resistant to platin salts who have not received more than 2 chemotherapy lines and have not been treated with bevacizumab or other VEGF inhibitors or other agents aiming at the VEGF receptor (in combination with paclitaxel, topotecan or pegylated liposomal doxorubicin until disease progression or unacceptable toxicity).

The reimbursement terms were negotiated based on a contract (managed entry agreement) between the Minister of Social Affairs and Public Health and the manufacturer after the regular procedure did not result in the drug entry in the reimbursement schedule (article 81 of the Royal Decree of 21 December 2001).[8] In theory, both financial-based agreements, performance-linked coverage and coverage with evidence development agreements are possible. Unfortunately, the appendices of such a contract, including e.g. a possible request for further evidence or results of price negotiations, are confidential.[9] However, research evaluating the first 71 approved contracts in Belgium found that the compensation mechanisms used in Belgium mainly exist out of a percentage of the declared turnover (84.6%) or a fixed amount per package (10.8%).[9] On 1 March 2014, a first 3-year contract was concluded for the first-line treatment of stage IV and the treatment of first recurrence in platinum-sensitive patients. A second 3-year contract was concluded on 1 July 2015 for the treatment of platinum-resistant patients. When these contracts expire, the manufacturer receives up to one extra year to submit a new application for regular reimbursement or to close a new confidential contract.

In an independent health technology assessment (HTA),[3] the safety, clinical effectiveness and cost-effectiveness of bevacizumab were assessed in two situations: first, in addition to first-line chemotherapy for ovarian cancer; and second, in the treatment of recurrent ovarian cancer (platinum-sensitive or platinum-resistant). The results should support the negotiations when a new reimbursement request is submitted to the Belgian authorities. In this article, we provide details and results of the health economic evaluation to answer the following question: what is the cost-utility of bevacizumab in these indications? The Consolidated Health Economic Evaluation Reporting Standards (CHEERS) checklist was used to optimise reporting of this evaluation (see S1 Table).[10]

## Methods

### Available RCTs

A systematic search of the clinical literature identified 5 relevant RCTs.[11–15] Results of the GOG-0213 trial[13] were only published in a meeting abstract at the time of the search and insufficient details were available to include this trial in the model. A brief description of the eligible population in the selected studies and the bevacizumab treatment schedule are provided in Table 1. Our model reflects the same patient population as in the underlying trial. In our model, a cohort of 60 years old women was modelled.

**Table 1:** Eligible patient population and bevacizumab treatment schedules in 4 peer-reviewed RCTs.

Setting	Study	Patients and bevacizumab schedule
**First-line**	**GOG-0218**[12] (1873 women) Median age: 60y	Previously untreated, incompletely resectable stage III or any stage IV ovarian cancer. Bevacizumab-initiation treatment: 15 mg/kg every 3 weeks for 5 cycles* Bevacizumab-throughout treatment: 15 mg/kg every 3 weeks for 21 cycles
	**Subgroup**:	Stage IV cancer.
	**ICON7**[14] (1528 women) Median age: 57y	Newly diagnosed ovarian cancer with stage IIb–IV or high-risk (grade 3 or clear cell histology) stage I–IIa disease. 7.5 mg/kg every 3 weeks for 5–6 cycles and continued for 12 additional cycles or until disease progression
	**Subgroup**:	Stage IV disease, inoperable stage III disease, or suboptimally debulked (>1 cm) stage III disease.
**Second-line**	**OCEANS**[11] (484 women) Mean age: 61y	Progression >6 months after completion of front-line platinum-based chemotherapy (platinum-sensitive). 15 mg/kg every 3 weeks
	**AURELIA**[15] (361 women) Median age: 61-62y	Progressed ovarian cancer within 6 months of completing >4 cycles of platinum-based therapy (platinum-resistant). 10 mg/kg every 2 weeks (or 15 mg/kg every 3 weeks in patients receiving topotecan in a schedule repeated every 3 weeks)

* The bevacizumab-initiation treatment was not modelled since the HR for OS was 1.078 (95% CI, 0.919 to 1.270), making the treatment on average more expensive and less effective expressed in LYG. There are also no good arguments to select this treatment arm if expressed in QALYs.

### General information

In accordance with the Belgian guidelines for economic evaluations,[16] the analysis is performed from the health care payer’s perspective and includes direct health care costs. Payments out of the government’s health care budget as well as patients’ co-payments and co-insurances are included. No extension to a societal perspective was modelled since there is no evidence for relevant differences in employment rate, transport or other costs. An incremental impact seems negligible and thus would not influence the results.

Bevacizumab in addition to standard chemotherapy is compared to standard chemotherapy alone (GOG-0218 and ICON7 trial: carboplatin and paclitaxel; OCEANS: carboplatin and gemcitabine; AURELIA: liposomal doxorubicin or paclitaxel or topotecan). Since bevacizumab is given in addition to standard chemotherapy, the focus is on the extra costs for this drug and related administration costs.

Bevacizumab might have an impact on mortality. Therefore, adopting a lifetime time horizon is necessary to capture the full possible impact on all relevant incremental costs and effects. In alternative scenarios, this time horizon is limited to 10 and 5 years. Costs and effects are discounted on a 3-weekly basis in the Markov model. The choice for 3-weekly cycles is determined by the quality of life (QoL) scenarios (see further). Half-cycle correction is applied. According to the Belgian guidelines for economic evaluations,[16] a yearly discount rate of 3% and 1.5% for costs and effects, respectively, is applied (or 0.1702% and 0.0857% 3-weekly). Following these guidelines,[16] scenario analyses are performed with a 0%, 3% and 5% discount rate for both costs and effects.

QoL is of major importance to patients with cancer and can be influenced by both disease and treatments. Therefore, a cost-utility analysis (CUA) is performed. As requested by the national guidelines, results of the cost-effectiveness analysis (CEA) in which the years of life are not adjusted for QoL are also presented. Both incremental costs (IC), incremental effects (IE) in life years gained and QALYs gained, and incremental cost-effectiveness ratios (ICER) will be presented separately in the results section.

The results of all identified RCTs where modelled separately because outcome definitions and treatment schedules were not comparable (see also below). We relied on published information since we did not have access to individual patient data. In case of multiple publications about a specific study, the most up-to-date data were used.

### Impact on mortality

Published Kaplan-Meier-curves (KM-curves) and hazard ratios for both overall survival (OS) and PFS were identified. OS and PFS were extracted from the KM-curves at fixed time points. This was done separately by two researchers using a different software (Datathief and R-Digitize). Because the difference between the two separate extractions was more than 1% in five cases of the 92 point estimates, a second check was performed and an incorrect measurement was excluded. Ultimately, the incremental difference between the control and bevacizumab group between the two researchers was always lower than 1%.

The mortality in the bevacizumab group was modelled through the hazard ratio (Table 2) (probability of death due to intervention = 1 − [probability of survival control therapy^(hazard ratio)]). Having no access to individual patient data, a constant hazard was assumed and the result was visually checked to see whether the modelled survival curves were in agreement with the extracted data from the published KM-curves. In the ICON7 high-risk group, this fit was not satisfying (the assumption of proportional hazards seems not justified) and both survival curves were therefore based on the extracted observations from the published KM-curves.

**Table 2:** OS hazard ratios.

	OS: mean (95%CI)
GOG-0218*[12]	0.885 (0.750–1.040)
GOG-0218 st.IV[17]	0.72 (0.53–0.97)
ICON7[14]	0.990 (0.850–1.140)
ICON7 HR[14]	0.780 (0.630–0.970)
OCEANS**[18]	0.960 (0.760–1.214)
AURELIA[15]	0.850 (0.660–1.080)

* GOG-0218: results of the updated analysis of August 26, 2011.

** OCEANS: results of the third interim analysis of March 30, 2012.

### Impact on progression-free survival

For PFS, the same approach as for OS was initially applied. Pooling the results of the GOG-0218 and ICON7 trials was considered inappropriate since different definitions for progression-free survival (PFS) were used and a less intensive treatment schedule was used in the ICON7 trial (7.5mg/kg vs 15mg/kg).[14]

The visual check on the modelled survival curves also showed that it was preferable to work with the extracted information from the published KM-curves. If PFS lines crossed each other, we conservatively decided to coincide the curves. This assumption was always in the advantage of the bevacizumab group (since the KM-curves showed a somewhat worse PFS after some time for the bevacizumab group).

We provide supplementary material with a presentation of the modelled OS and PFS curves and the observed OS and PFS at fixed points in time extracted from the published KM-curves (S1 Fig). This provides the readers a view on the modelled lifetime horizon and also allows a visual validation of the models.

### Extrapolation

KM-curves were published with a time window of 30 months (AURELIA) up to 60 months (ICON7) for OS and 12 months (AURELIA) up to 60 months (ICON7) for PFS. The observed differences in OS and PFS do not disappear at once after these follow-up periods. Therefore, extrapolation is appropriate. The 3-weekly probabilities during the last year in the trial are used to extrapolate. For the AURELIA trial, extrapolation was based on the last 6 months for both OS and PFS. This was also the case for PFS in the OCEANS trial. In the AURELIA trial, for overall survival, only 12 and 13 patients were at risk in the placebo and bevacizumab treatment arm, respectively, at 30 months follow-up. This might result in great uncertainty, which is then also used in the extrapolation phase. However, the 3-weekly mortality over a six-month period did not vary much when modelling up to 18, 24 or 30 months (between 4.41% and 4.46%). In the GOG, ICON7, and OCEANS trial, at least 56, 117, and 23 patients were at risk, respectively, at the last follow-up moment.

Three possible extrapolation scenarios are applied: 1) the 3-weekly risk of death remains constant over time (exponential survival); 2) the 3-weekly mortality risk increases with the absolute increase in mortality risk of the general Belgian female population of the same age; 3) the 3-weekly mortality risk increases with the relative increase in this mortality risk of the general Belgian female population of the same age. A first view on results showed that the first two options do not differ much. The third option results very probably in too pessimistic survival curves. Therefore, in the base case scenario, the first most conservative extrapolation approach was applied. The other two options were modelled in scenario analyses.

### Impact on quality of life

The effect of first-line bevacizumab on QoL was reported in the GOG-0218 trial (measured with the FACT-O TOI instrument) and ICON7 trial (measured with the EORTC QLQ-C30 and QLQ-OV28 instruments). The results show an early (18 weeks) worsening in QoL when using bevacizumab. This negative effect disappeared in the long run in the GOG-0218 trial (60 weeks), but not in the ICON7 trial (54 weeks).[3] With second-line bevacizumab fewer patients reported abdominal/gastrointestinal symptoms (e.g. pain, ascites …) during chemotherapy, measured with EORTC QLQ-OV28. However, no differences in QoL were found when using other instruments (FOSI, EORTC QLQ-C30 and FACT-O-TOI).[3] Unfortunately, no utility values were reported (see Discussion).

In the NICE TA284 manufacturer submission (first-line bevacizumab),[19] QoL data from the ICON7 trial, measured with the generic EQ-5D questionnaire, were presented. The manufacturer’s submission did not present the utilities per treatment arm. In contrast, these values were aggregated for the PFS and progressed disease (PD) health states (see Table 3). As such, an improvement in QoL was modelled indirectly. In contrast with the available evidence, no decrease in QoL during the first cycles with bevacizumab was modelled. In case of the TA285 manufacturer submission (second-line bevacizumab), the values were retrieved from other studies and the manufacturer noticed that “*the use of utility data from OVA-301 presented in TA222 should be interpreted with caution due to little overlap in the types of adverse event between OVA-301 and OCEANS*.”[18]

**Table 3:** QoL values.

3-weekly cycles	Base case	Values from TA285[18]	Values from TA284*[19]
**Progression-free survival**			
Cycle 1	Mean 0.72 (beta-distribution, min: 0.62; max: 0.82) for all PFS cycles.	Mean 0.718(beta-distribution, 2.5%: 0.699–97.5%: 0.737) for all PFS cycles.	Mean: 0.6571 (SD: 0.0133)**
Cycle 2			Mean: 0.7153 (SD: 0.0118)
Cycle 3			Mean: 0.7443 (SD: 0.0110)
Cycle 4			Mean: 0.7683 (SD: 0.0100)
Cycle 5			Mean: 0.7643 (SD: 0.0112)
Cycle 6			Mean: 0.7444 (SD: 0.0121)
Cycle 7			Mean: 0.7444 (SD: 0.0121)
Cycle 8			Mean: 0.7638 (SD: 0.0131)
Cycle 9			Mean: 0.7638 (SD: 0.0131)
Cycle 10			Mean: 0.7718 (SD: 0.0129)
Cycle 11			Mean: 0.7718 (SD: 0.0129)
Cycle 12			Mean: 0.7638 (SD: 0.0136)
Cycle 13			Mean: 0.7638 (SD: 0.0136)
Cycle 14			Mean: 0.7785 (SD: 0.0155)
Cycle 15			Mean: 0.7785 (SD: 0.0155)
Cycle 16			Mean: 0.7533 (SD: 0.0165)
Cycle 17			Mean: 0.7533 (SD: 0.0165)
Cycle 18			Mean: 0.7760 (SD: 0.0170)
Cycle 19 and further			Mean: 0.8129 (SD: 0.0113)
**Progressed disease**	Idem as utility PFS to model no difference between the two treatment arms	Mean 0.649 (beta-distribution, 2.5%: 0.611–97.5%: 0.686)	0.7248 (fixed)

* QoL values from a study with a lower dose of bevacizumab administered (7.5mg/kg).

** these values are also modelled with a beta probability distribution.

In our base case scenario, we conservatively assume no decrease in QoL due to bevacizumab treatment and model an equal QoL through all cycles. We assume a utility value of 0.72 (as in PFS in TA285 and PD in TA284) with an uncertainty ranging from 0.62 to 0.82 (minimum and maximum values in the NICE submissions, modelled with a beta-distribution). In two scenario analyses, we model the input from the two manufacturer’s submissions to see in how far this influences our results (see Discussion) (Table 3).

### Costs drug treatment

The incremental bevacizumab treatment costs were calculated by estimating the bevacizumab drug cost per cycle, the number of bevacizumab treatment cycles, and adding the extra costs for administration.

The bevacizumab drug cost per cycle is based on the Belgian sample of ovarian cancer patients diagnosed between 2008 and 2013 receiving bevacizumab, transmitted by the Belgian Cancer Registry. The year of costs is mainly 2014 since the majority of the patients (82.2%) received their first session in 2014. We remark that only patients with reimbursed bevacizumab treatment were selected for this estimate since, based on expert opinion, it is possible that a lower dose is administered when the treatment is not reimbursed. For Belgian ovarian cancer patients, the average bevacizumab cost was €3169 (SD: €977, based on 806 treatment cycles).[3] Applying the central limit theorem, a normal distribution is applied to include the average cost per bevacizumab treatment cycle. In Belgium, the price of a 100mg vial and 400mg vial is €333.67 and €1240.65, respectively. With a 15mg/kg body weight bevacizumab administration, the average cost of €3169 corresponds to an average population weight of about 64kg. The small difference in drug waste is taken into account when a different dose is administered, which resulted in an average bevacizumab treatment cost per cycle of €1676.2 (7.5mg/kg) and €2176.8 (10mg/kg) for the ICON7 and AURELIA model, respectively.

The bevacizumab cost per cycle is multiplied with the number of treatment cycles (total drug cost in Table 4). For the GOG-0218, ICON7 and OCEANS trial, the average number of treatment cycles are provided in the NICE manufacturer submissions (see Table 4). For the high-risk subgroup of the ICON7 trial no separate information on the mean treatment duration could be retrieved. In case of the AURELIA trial, only the median treatment duration was published: three cycles (range, one to 17 cycles) in the CT arm versus six cycles (range, one to 24 cycles) in the bevacizumab arm.[15] Treatment exposure was also published in a figure. With the data extraction software Datathief, an average treatment duration of 6.6 cycles of 4 weeks was extracted for the bevacizumab group and 4.2 cycles for the chemotherapy treatment arm. With a 4-weekly treatment cycle, this results in an average treatment duration of 26.2 and 16.7 weeks.[3] In case of the stage IV subgroup of the GOG-0218 trial, the manufacturer’s submission to the National Institute for Health and Disability Insurance (NIHDI) mentions an average treatment duration of 11.9 cycles (35.7 weeks), which is two cycles less than in the overall GOG-0218 population (Table 4). It is not clear whether the average chemotherapy treatment duration was also shorter. Conservatively in favour of bevacizumab treatment, the extra number of administrations was also reduced with two cycles.

**Table 4:** Mean treatment duration and bevacizumab treatment costs.

	GOG-0218[19]	GOG-0218 st.IV	ICON7[19]	OCEANS[18]	AURELIA**[15]
**Mean treatment duration**					
**Bevacizumab**	41.93 weeks	35.7 weeks*	42.99 weeks	50.74 weeks	6.55 cycles of 4 weeks
**Chemotherapy**	16.55 weeks	NA	15.96 weeks	22.50 weeks	4.16 cycles of 4 weeks
**Bevacizumab treatment costs**					
**Total drug costs**	€44 286	€37 706	€24 020	€53 591	€28 529
**Extra administration costs**	€2870***	€2165	€3057	€3193	€1622****

* Source: INAMI—Service des Soins de Santé, Rapport jour 60, AVASTIN 25 mg/ml solution à diluer pour perfusion.

** Numbers were extracted from figure 4 in the original publication of Pujade et al.[15]

*** €2870 = €339.24 x (41.93 weeks– 16.55 weeks)/3-weekly cycle.

**** €1622 = €339.24 x 2.39 cycles of 4 weeks x 2 (i.e. 2-weekly bevacizumab administration).

Next to the drug cost, all extra costs on the day of administration are also taken into account. These costs are based on Belgian reimbursement data (from the InterMutualistic agency—IMA). When bevacizumab was administered without other chemotherapy, the average cost on the day of treatment was €339 (N = 423, SD: 204.25).[3] Applying the central limit theorem, this was modelled with a normal distribution. This extra cost on the day of administration was only counted for the extra treatment duration after chemotherapy (Table 4). To avoid complexity, these bevacizumab treatment costs are allocated to all patients at the first cycle of the model.

Since bevacizumab is an add-on therapy, no differences in chemotherapy costs were included for the bevacizumab treatment arm versus the chemotherapy arm.

### Costs adverse events

A systematic review of the clinical literature[3] showed an increase in adverse events (AEs) in the bevacizumab treatment arm. We tried to include the AEs with a possible significant impact on costs. Four AEs were selected of which the absolute difference in incidence was relatively large or because the cost of treating the AE was potentially high: hypertension grade 2+, gastrointestinal (GI) perforation grade 2+, venous thromboembolism grade 3+ and arterial thromboembolism any grade. Hypertension was identified in the IMA data based on the reimbursement of the following pharmaceutical products: ATC C02 (antihypertensives), ATC C03 (diuretics), ATC C07 (beta blocking agents), ATC C08 (calcium channel blockers) or ATC C09 (agents acting on the renin-angiotensin system). Of bevacizumab patients, 92.1% had at least one of these products reimbursed following their diagnosis of ovarian cancer. However, only 4.6% of bevacizumab patients started with any of these products after their first bevacizumab session. For this reason and the fact that it is difficult to differentiate the hypertension grades in the available data, no cost was retained for hypertension. This is a conservative approach not disfavouring bevacizumab. Similarly, for thrombosis, the IMA data showed that 98.7% of bevacizumab patients had reimbursements for ATC B01A (antithrombotic agents) pharmaceutical products. All of these patients started with antithrombotic agents prior to their bevacizumab treatment. Therefore, no cost was retained for thrombosis. This is also a conservative approach not disfavouring bevacizumab.

Finally, only a cost for GI perforations was included. The number of events was modelled through a beta distribution (control group: 13/2095; bevacizumab group: 36/2109).[3] The cost for GI perforations was retrieved from the Technical cell (TCT) database that contains the administrative, inpatient and day-care hospital stays in Belgian general hospitals. In our sample of ovarian cancer patients (2008–2013), twelve inpatient hospital stays were identified with ID-9-CM codes 569.83 (perforation of the intestine) or 569.81 (fistula of intestine, excluding rectum and anus) as the principal diagnosis of the stay. The mean cost was €10 458 (SD: 7778).[3] Because the costs are based on a very small sample and costs for AEs are usually skewed to the right, they are represented by a gamma-distribution reflecting the same mean, standard deviation and minimum of our small sample (Gamma distribution: alpha: 1.23; beta: 7016.81; shift: 1836).

No differences in follow-up were modelled since it is not expected that these costs are very different between patients with and without bevacizumab treatment.

### Uncertainty

Probabilistic sensitivity analyses (PSA) and one-way scenario analyses were performed in Microsoft Excel 2013, using @Risk software (Palisade Corporation). Scenario analyses included the following: population (GOG-0218, GOG-0218 stage IV, ICON7, ICON7 high-risk, OCEANS, and AURELIA); time horizon (lifetime (base case), 10 years, 5 years); discount rate (3% for costs and 1.5% for effects (base case), and 0%, 3%, or 5% for both costs and effects); extrapolation (constant mortality (base case), absolute or relative increase (see above)); QoL (equal QoL in both treatment arms (base case), values from manufacturer submissions TA284 or TA285). Because of the confidential price agreements, sensitivity analyses were also performed with price discounts between 0% and 100%.

Results are presented in table format (mean and 95% credibility intervals) and figures. Both cost-effectiveness planes, cost-effectiveness acceptability curves, and tornado graphs are provided.

## Results

Table 5 gives an overview of the base case results for all modelled trials. In first line, the ICER is on average €158 000/QALY (GOG-0218) or €443 000/QALY (ICON7). In the most favourable subgroup analyses of these trials the ICERs improved to about €52 000/QALY (GOG-0218 stage IV subgroup) or €82 000/QALY (ICON7 high-risk subgroup). In second line, the average ICERs were €587 000/QALY (OCEANS) or €172 000/QALY (AURELIA).

**Table 5:** IC, IE & ICERs for the base case scenario (GOG-0218 (st.IV), ICON7 (high risk), OCEANS, and AURELIA).

	GOG-0218	GOG-0218 st.IV	ICON7	ICON7 HR*	OCEANS	AURELIA
	mean	mean	mean	mean	mean	mean
	(2.5%–97.5%)	(2.5%–97.5%)	(2.5%–97.5%)	(2.5%–97.5%)	(2.5%–97.5%)	(2.5%–97.5%)
Life expectancy (years)						
Control	4.06	3.27	6.24	3.66	3.66	1.46
Bevacizumab	4.48	4.34	6.33	4.12	3.80	1.70
	(3.94–5.07)	(3.36–5.53)	(5.61–7.08)	(4.12–4.12)	(3.17–4.53)	(1.37–2.10)
QALYs (years)						
Control	2.93	2.36	4.49	2.63	2.64	1.05
	(2.60–3.25)	(2.09–2.62)	(3.99–5.00)	(2.34–2.93)	(2.34–2.94)	(0.93–1.17)
Bevacizumab	3.22	3.13	4.56	2.97	2.74	1.23
	(2.71–3.82)	(2.36–4.04)	(3.81–5.32)	(2.63–3.30)	(2.20–3.37)	(0.95–1.56)
IC	€ 47 268	€ 39 984	€ 27 188	€ 27 188	€ 56 897	€ 30 259
	(€46 309–€48 209)	(€39 149–€40 786)	(€26 611–€27 743)	(€26 611–€27 743)	(€55 750–€58 022)	(€29 611–€30 880)
IE (LYG)	0.42	1.07	0.09	0.46	0.13	0.24
	(-0.12–1.01)	(0.09–2.25)	(-0.63–0.84)	(0.46–0.46)	(-0.50–0.87)	(-0.09–0.64)
IE (QALY gained)	0.30	0.77	0.06	0.33	0.10	0.18
	(-0.09–0.73)	(0.07–1.62)	(-0.45–0.60)	(0.29–0.37)	(-0.37–0.63)	(-0.07–0.47)
ICER (€/LYG)**	*€ 113 523*	*€ 37 299*	*€ 314 963*	€ 59 008	*€ 424 318*	*€ 124 317*
				(€57 755–€60 212)		
ICER (€/QALY gained)**	*€ 157 816*	*€ 51 931*	*€ 443 027*	€ 82 277	*€ 587 182*	*€ 172 370*
				(€73 597–€92 577)		

There is no confidence interval for the life expectancy in the control arm because it was modelled deterministically.

* we remark that the modelling of the ICON7 high-risk subgroup, making use of the hazard ratios, did not provide a good fit with the point estimates of the published KM-curve (see ‘validation of modelling outcomes’ in the full repor[3]). Therefore, it was decided to model survival through the fixed points in time extracted from the published KM-curves. This is rather a deterministic approach to model overall survival and results in a too narrow credibility interval around the ICERs.

** Calculation of the average ICER based on the 1000 simulations is not reliable if the outcomes are situated in different quadrants. In these cases, the presented ICERs are calculated by dividing the mean incremental cost by the mean incremental benefit.

**Fig 1:**
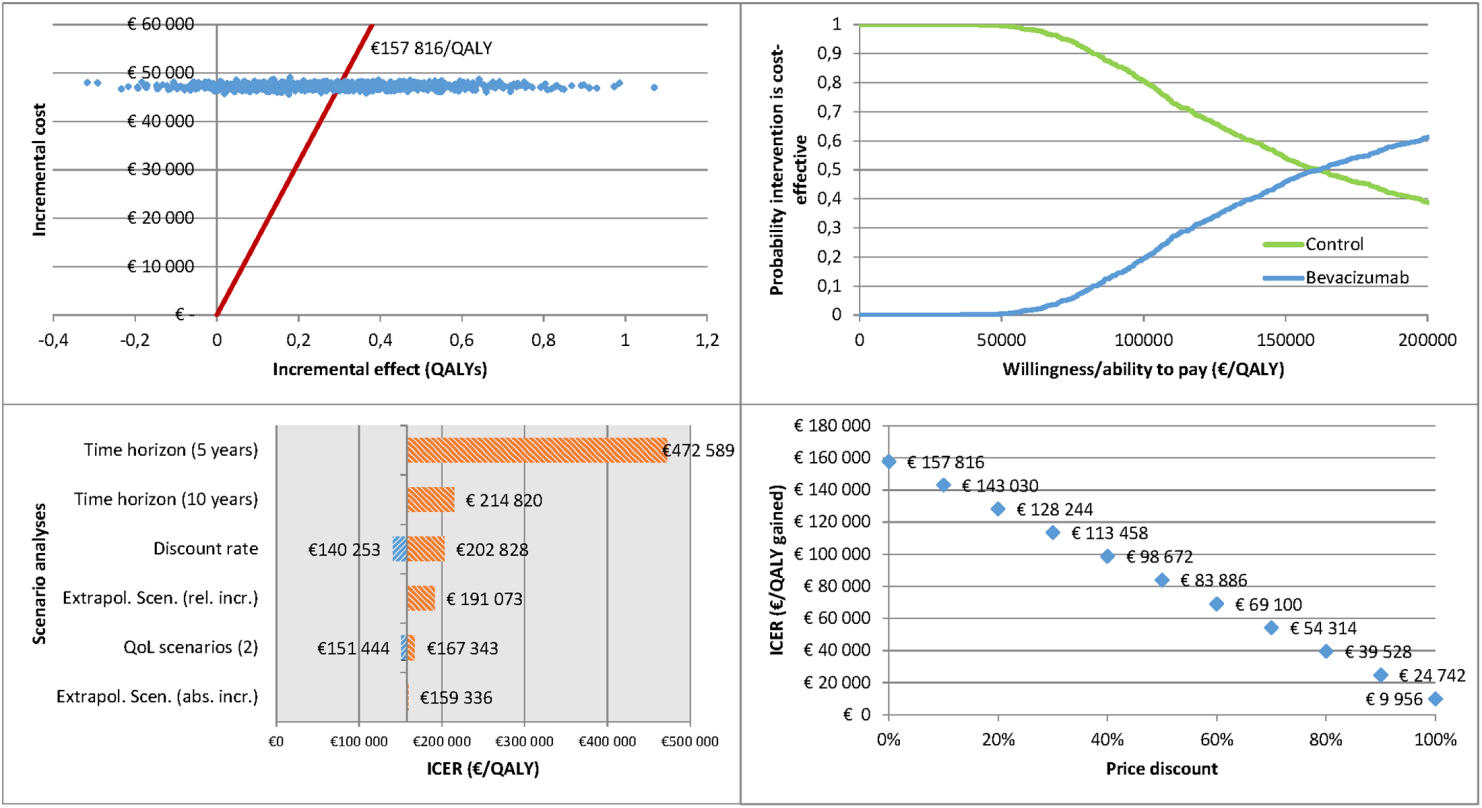
Results of the economic evaluation for the GOG-0218 trial.

Fig 1 shows the cost-effectiveness plane, cost-effectiveness acceptability curve (CEAC), tornado graph, and price discount scenarios for the GOG-0218 trial. The high uncertainty around the treatment effect is reflected by the wide confidence interval around the incremental effects and part of the simulations situated in the fourth quadrant of the cost-effectiveness plane. The CEAC shows that up to a willingness-to-pay (WTP) of €50 000/QALY there is a 0% probability that bevacizumab is considered a cost-effective intervention. The tornado graph and price discount scenarios indicate the results are most sensitive to both the price of bevacizumab and the time horizon. The alternative QoL scenarios, based on the values from the manufacturer’s submissions to NICE, do not have a major impact on results. An 80% discount is needed to reach an average ICER of about €40 000/QALY or even 90% to reach an average ICER of about €25 000/QALY.

Similar information is available for all the other modelled trial results in supplementary material (S2 Fig).

## Discussion

The calculated ICERs for bevacizumab treatment versus standard chemotherapy alone for ovarian cancer patients are relatively high in both the first- and second-line setting. The health gains seem to be relatively small in relation to the extra costs associated with bevacizumab treatment. In first line, the average ICERs are €158 000/QALY (GOG-0218) and €443 000/QALY (ICON7). Results are more positive in subgroups of these trials. The most optimistic outcomes are based on the stage IV subgroup of the GOG-0218 trial. In this subgroup analysis, average ICERs amount to €52 000/QALY in a rather optimistic scenario in which no decrease in QoL is assumed (see further), results are extrapolated to a lifetime time horizon with a constant mortality, and conservative assumptions for not including costs for specific side effects are made. In second line, the average ICERs based on the OCEANS and AURELIA trials amount to €587 000/QALY and €172 000/QALY, respectively.

A systematic review[3] of previously published economic evaluations identified 11 relevant publications.[18–30] Two manufacturer submissions published by NICE[18 19] were included. The related Evidence Review Group (ERG) evaluations were also consulted.[20 21] These four publications were counted as two economic evaluations. For a detailed overview we refer to the full HTA report.[3] Economic evaluations referring to the GOG-0218[19, 25, 26, 29, 30] and/or ICON7[19, 22, 30] trials concluded that bevacizumab was not cost-effective. Of the four evaluations modelling the high-risk subgroup of the ICON7 trial,[22, 23, 27, 28] only two formulated rather positive conclusions by applying relatively high WTP values: Duong et al.[27] considered bevacizumab cost-effective in 56% of tested scenarios at a threshold of CAD100 000 (~€66 000) per QALY; and Chan et al.[23] considered an ICER of approximately $170 000 (~€152 000) per LYG near cost-effective based on current benchmarks. In the latter case, the authors referred to previous reimbursement decisions. However, it should be taken into account that it’s not clear in how far economic considerations were taken into account in previous policy decisions. As such, it is possible that inefficiencies from the past persist. In second line, based on the AURELIA trial, Chappell et al.[24] use a WTP threshold of $100 000 (~€89 000) per progression-free life-year saved to demonstrate bevacizumab is cost-effectiveness. However, PFS is not correlated with overall survival and extra costs per progression-free life-year are difficult to interpret. Even in the manufacturer’s submission file to NICE, the ICER of bevacizumab at the licensed dose was by far higher than NICE’s threshold of £20 000 to £30 000 (~€23 000 to ~€34 000) per QALY. In these files of the manufacturer, the probabilistic ICER was on average £145 000[19] (~€166 000) per QALY based on the GOG-0218 study (first line) and £222 000[18] (~€254 000) per QALY based on the OCEANS study (second line). Furthermore, in the latter case, as noticed by the Evidence Review Group, the manufacturer modelled a positive impact on OS based on the September 2010 results, which was not sustained in the March 2012 analysis of this trial. Finally, we remark that none of the previous published evaluations modelled the stage IV subgroup of the GOG-0218 trial.

No good QoL estimates for both the control arm and bevacizumab treatment arm were identified in the economic evaluations. A lot of different assumptions were made related to the impact of bevacizumab treatment on QoL, both in favour and disfavour of the drug. Some authors did not include QoL,[23, 25] others assumed an equal impact on QoL in both arms,[22, 24] also a decrease in QoL was assumed during bevacizumab treatment[26, 30] or due to more side effects, while most modelled an improvement thanks to an improved PFS.[18, 19, 27–30] First, we remark that the clinical evidence in the first-line setting shows an early (18 weeks) worsening in QoL which might disappear in the long run (see above). If this is not taken into account, results can be considered being optimistic. Second, the ICON7 protocol incorporated the EQ-5D questionnaire to measure patients’ HRQoL.[31] Yet, the article entitled “*Standard chemotherapy with or without bevacizumab in advanced ovarian cancer*: *quality-of-life outcomes from the International Collaboration on Ovarian Neoplasms (ICON7) phase 3 randomised trial*”[32] did not publish these values. They were only published in the manufacturer’s submission to NICE. Unfortunately, results are not provided per treatment arm but only per health state (progression-free or progressed disease). As such, the size of the possible negative short-term impact on QoL is not clear. On the contrary, only a positive effect through longer PFS is modelled. Furthermore, as previously mentioned by the Evidence Review Group, the ICON7 trial “*employed a lower dose of bevacizumab than in the NICE scope*. *Any AEs caused by the higher dose of bevacizumab as specified in the NICE scope would not be captured using the utility data from the ICON7 trial*.”[20] Applying utility estimates from the ICON7 trial to the GOG-0218 trial might thus be too optimistic.

Another point of attention is the choice of the primary endpoint. Surrogate endpoints are frequently used in oncology clinical trials and also in this case the trials were powered to find a difference in PFS instead of OS. According to the EUnetHTA guidelines on clinical endpoints used for relative effectiveness assessment: “*If progression-free survival (PFS) is used as an endpoint there should be sufficient independent evidence to demonstrate that this is associated with overall survival*. *… Overall survival is the gold standard for demonstrating clinical benefit and as such should be used where possible*. *… In the metastatic setting*, *data on PFS alone is insufficient and should be coupled with quality of life assessment and survival data*, *the maturity of which will be considered on the case by case basis*.”[33] A systematic review has tried “*to identify and evaluate trial-level meta-analyses of randomized clinical trials quantifying the association between a surrogate endpoint and overall survival in medical oncology*. *Trial-level correlations test whether treatments that improve the surrogate endpoint also improve the final endpoint and are widely considered the strongest evidence to validate a surrogate endpoint*.”[34] Unfortunately, there was only a low correlation and it was concluded that the evidence supporting the use of surrogate endpoints in oncology was limited.[34] In the metastatic setting, the study identified 8 meta-analyses examining whether gains in PFS predict overall survival in metastatic breast cancer. Six reported a low correlation; 1 reported a medium correlation; and only 1 reported a strong correlation.[34] In a US study,[35] Kim and Prasad identified 54 approvals made during their search period, with 36 drugs (67%) approved on the basis of a surrogate endpoint. In 19 cases (53%), rate of response, measured by a reduction in tumour size or volume, was the primary measure of efficacy. In the other 17 cases (47%), PFS or disease-free survival was used. With a median follow-up of 4.4 years, 5 drugs were subsequently shown to improve overall survival in randomized studies, 18 drugs failed to improve overall survival as primary or secondary outcomes, and 13 drugs continue to have unknown survival effects.[35] Based on available evidence, the use of PFS is problematic and HTA researchers prefer to focus on the outcomes that really matter to the patient, being overall survival and overall QoL. In most countries, the preferred outcome measure in HTA are life-years and QALYs.[36] Also the Belgian guidelines recommend to use these outcomes instead of intermediary outcomes.[16] To measure the impact on QoL, the EUnetHTA guidelines on HRQoL recommend to include a generic utility instrument in complement to the disease-specific instruments.[37] This would contribute to the quality and reliability of economic evaluations.

Our economic evaluation is the first one to calculate the ICER for the stage IV subgroup of the GOG-0218 trial. One of the major reasons to do so is because this indication is currently reimbursed in Belgium through a confidential contract between the Minister and the manufacturer (managed entry agreement). The dangers of such analysis are well-known.[3] In 2016, regarding the subgroup analyses of the GOG-0218 trial, NICE considered that “*the results from GOG-0218 (Randall 2013) represent an exploratory subgroup analysis of people with stage IV disease; confirmatory studies are required to strengthen Randall et al*.*’s conclusion that bevacizumab is more effective in people with stage IV disease*. *In addition*, *this analysis may not address the uncertainty in the survival benefit of bevacizumab*. *Considering these limitations*, *and the high incremental cost effectiveness ratio (ICER) for bevacizumab in the overall patient population*, *this exploratory subgroup analysis does not warrant a review of the guidance*”.[38] Currently, at least three studies are ongoing in first line that include stage IV patients (NCT01462890 (BOOST); NCT01081262 (GOG-0241); and ISRCTN10356387 (ICON8B)). The results of these studies should be followed up.

Finally, evidence based medicine “*integrates the best external evidence with individual clinical expertise and patients’ choice*.”[39] Havrilesky et al.[40] studied patient preferences for seven attributes: mode of administration, visit frequency, peripheral neuropathy, nausea and vomiting, fatigue, abdominal discomfort and PFS. Ninety-five women with advanced or recurrent ovarian cancer participated. PFS was found to be the predominant driver of patient preferences for chemotherapy regimens, but overall survival and quality of life were not included in the experiment. Furthermore, patients’ choices indicated that they were willing to accept a shorter PFS to avoid severe side effects: a reduction of 6.7 months to reduce nausea and vomiting from severe to mild, 5.0 months to reduce neuropathy from severe to mild, and 3.7 months to reduce abdominal symptoms from severe to moderate.[40] In a larger study,[41] 1413 women with ovarian cancer participated in a survey about their preference regarding side effects and therapy endpoints. “*Patients were willing to accept higher toxicity for a greater OS improvement*, *but not for attainment of PFS gains or stable disease*. *… Notable was the decreased acceptance of toxicity and daily impairment in the setting of recurrence*.”[41] Although it is unfeasible to include the individual patient’s preference in general reimbursement decisions, it is still of absolute importance to consider this when choosing a specific treatment option.

In conclusion, the incremental cost-effectiveness ratio of bevacizumab for the treatment of ovarian cancer is relatively high, both in first- and second-line setting. In Belgium, bevacizumab is currently reimbursed in three indications through confidential contracts. New negotiations will probably be performed in the beginning of 2018. We recommend to take into account both the clinical and health-economic findings in the decision-making process on the (further) reimbursement of bevacizumab.

## Supporting information

S1 Table CHEERS checklist. (DOCX)

S1 Fig Modelled OS and PFS curves. (DOCX)

S2 Fig Figures with results for other modelled RCTs. (DOCX)
