# Supplementary material for: First- and second-line bevacizumab in ovarian cancer: A Belgian cost-utility analysis
Source: PLoS One. 2018 Apr 9;13(4):e0195134. doi: 10.1371/journal.pone.0195134 (PMC5891000; doi:10.1371/journal.pone.0195134)

Figure 1 – Presentation of modelled OS and PFS curves and extracted KM point estimates allowing visual validation of the models


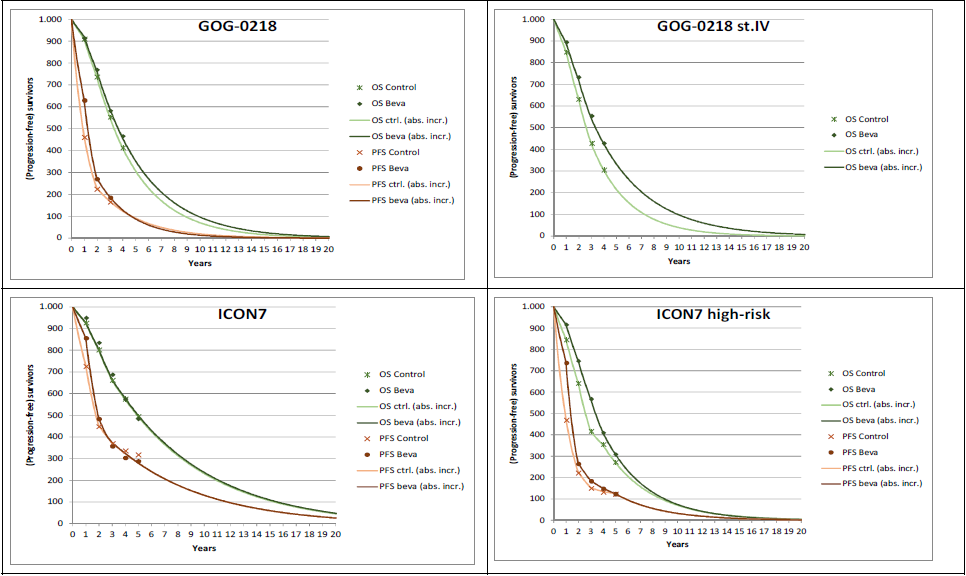


Figure 2 – Presentation of modelled OS and PFS curves and extracted KM point estimates allowing visual validation of the models


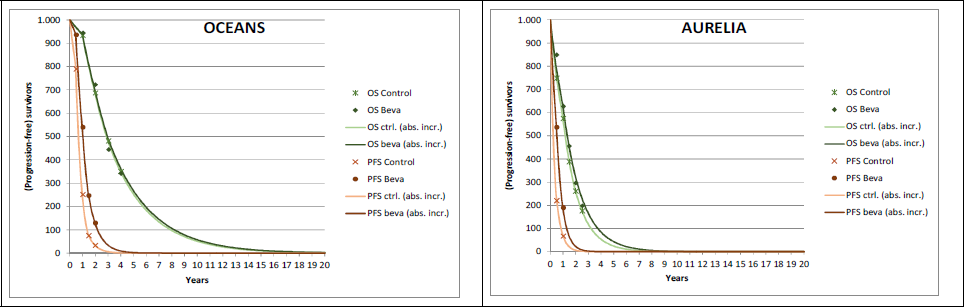

Supplement: S1 Fig — (DOCX) [file pone.0195134.s002.docx]
