# Supplementary material for: First- and second-line bevacizumab in ovarian cancer: A Belgian cost-utility analysis
Source: PLoS One. 2018 Apr 9;13(4):e0195134. doi: 10.1371/journal.pone.0195134 (PMC5891000; doi:10.1371/journal.pone.0195134)

Figure 1 – Results of the economic evaluation for the GOG-0218 trial (stage IV subgroup)


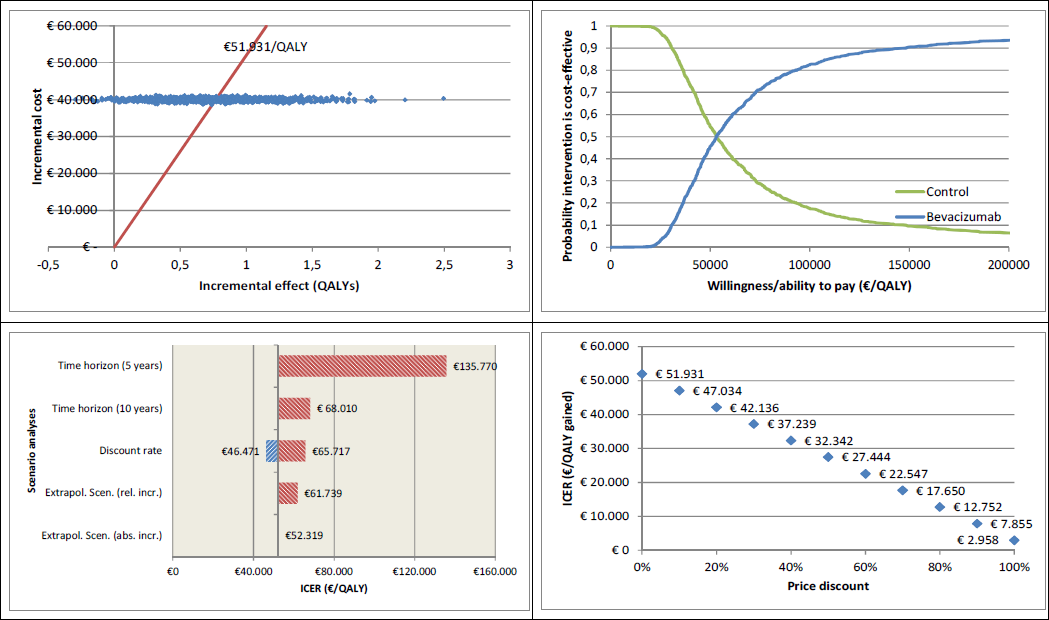


Figure 2 – Results of the economic evaluation for the ICON7 trial


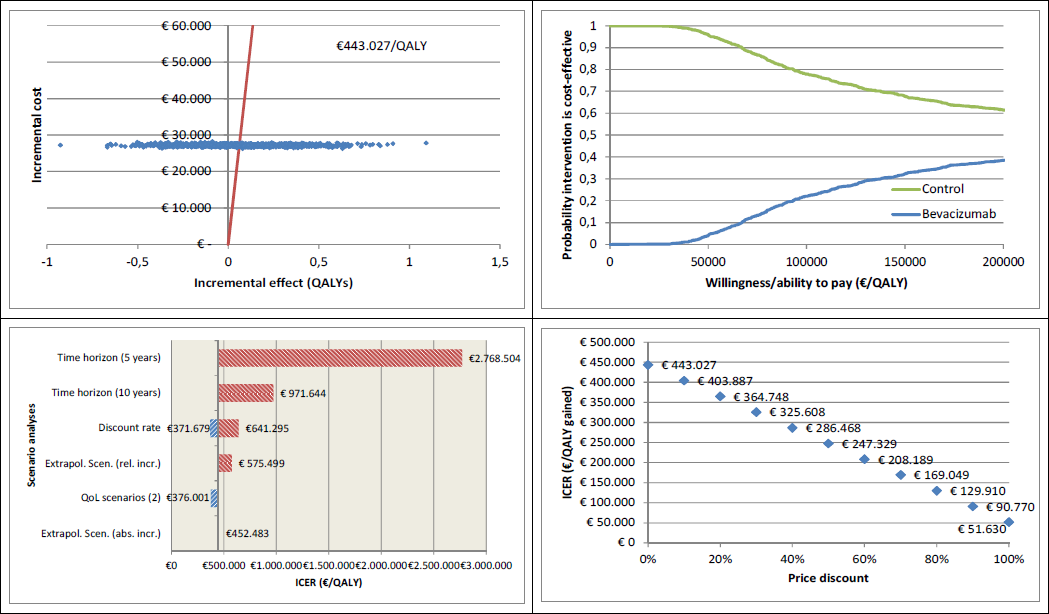


Figure 3 – Results of the economic evaluation for the ICON7 trial (high-risk subgroup)


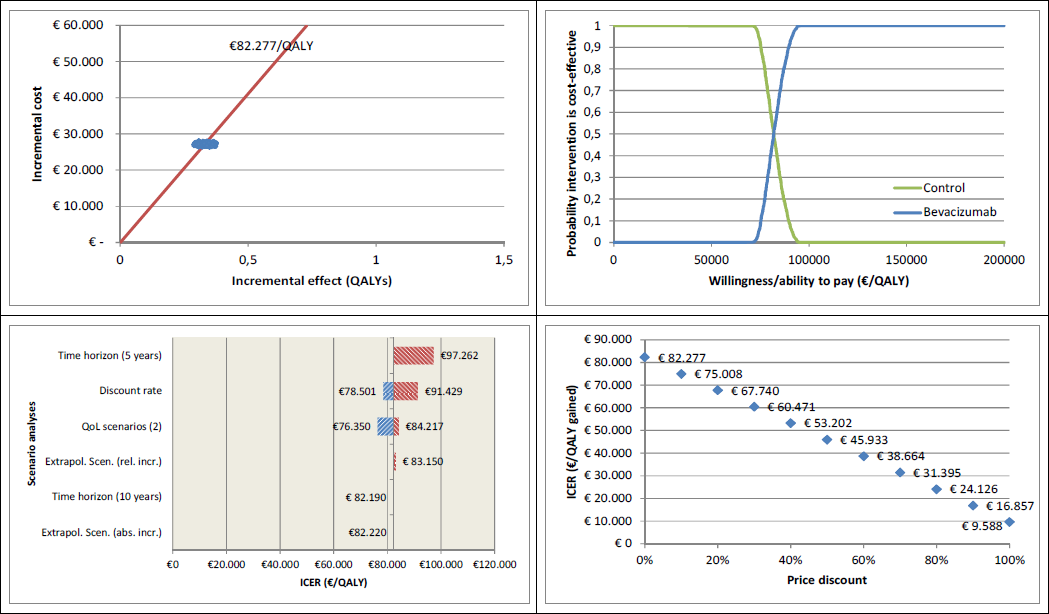

*In this scenario, modelling through the hazard ratios did not provide a good fit with the published evidence (see ‘validation of modelling outcomes’ in the full report). Therefore, a deterministic approach was modelled which results in an underestimation of modelled uncertainty (i.e. the cloud of simulated dots on the cost-effectiveness plane is too narrow).*

Figure 4 – Results of the economic evaluation for the OCEANS trial


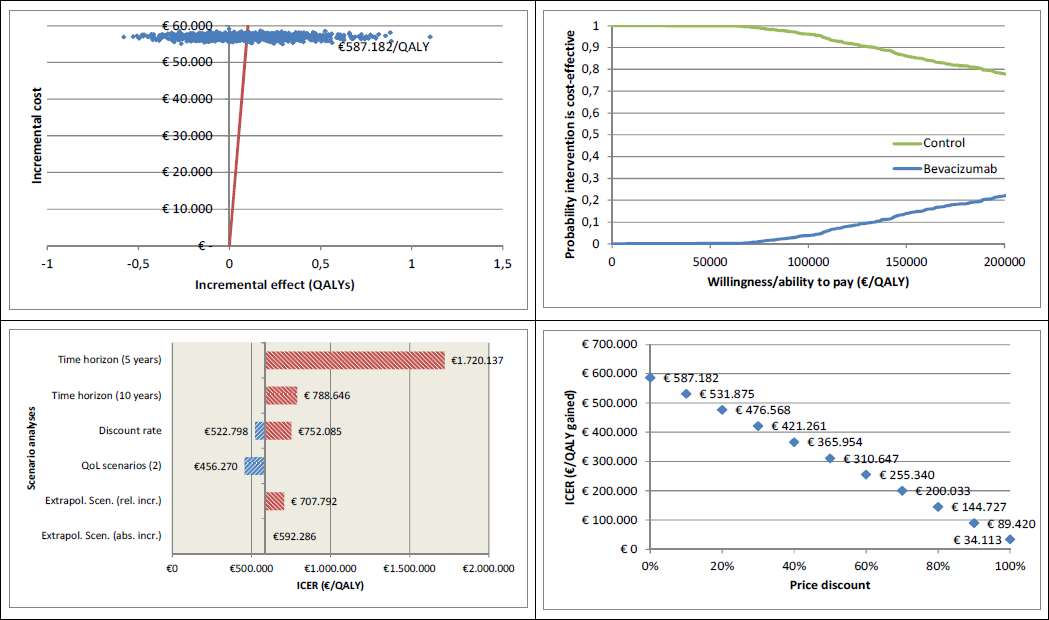


Figure 5 – Results of the economic evaluation for the AURELIA trial


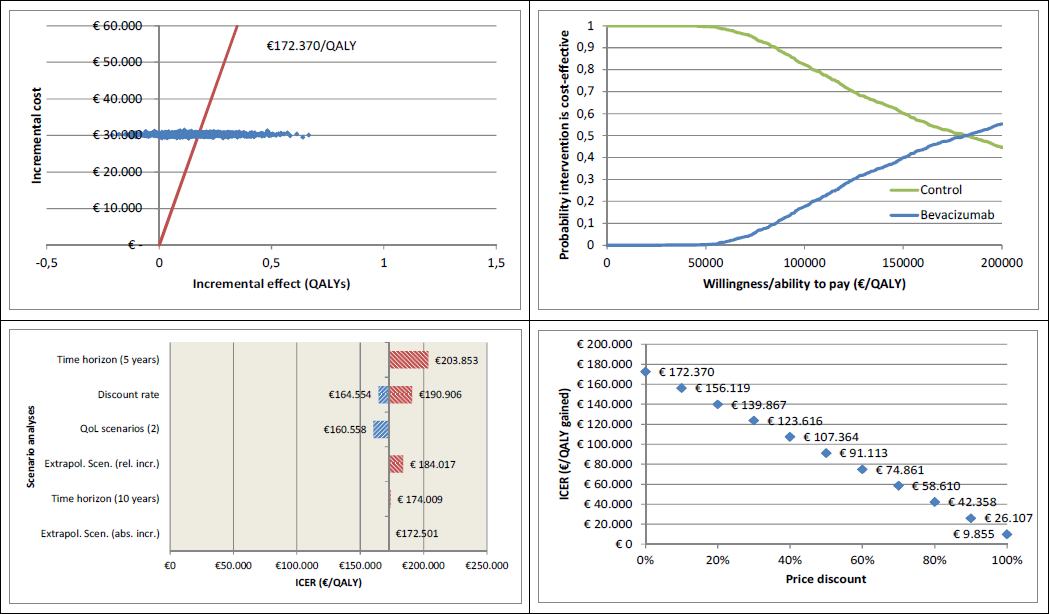

Supplement: S2 Fig — (DOCX) [file pone.0195134.s003.docx]
